# Initial intramuscular perfusion pressure predicts early skeletal muscle function following isolated tibial fractures

**DOI:** 10.1186/1749-799X-3-14

**Published:** 2008-04-17

**Authors:** Michael Müller, Aleaxander C Disch, Nicole Zabel, Norbert P Haas, Klaus D Schaser

**Affiliations:** 1 Charité – University Medicine Berlin, Center of Musculoskeletal Surgery, Berlin, Germany

## Abstract

**Background:**

The severity of associated soft tissue trauma in complex injuries of the extremities guides fracture treatment and decisively determines patient's prognosis. Trauma-induced microvascular dysfunction and increased tissue pressure is known to trigger secondary soft tissue damage and seems to adversely affect skeletal muscle function.

**Methods:**

20 patients with isolated tibial fractures were included. Blood pressure and compartment pressure (anterior and deep posterior compartment) were measured continuously up to 24 hours. Corresponding perfusion pressure was calculated. After 4 and 12 weeks isokinetic muscle peak torque and mean power of the ankle joint in dorsal and plantar flexion were measured using a Biodex dynamometer.

**Results:**

A significant inverse correlation between the anterior perfusion pressure at 24 hours and deficit in dorsiflexion at 4 weeks was found for both, the peak torque (R = -0.83; p < 0.01) and the mean power (R = -0.84; p < 0.01). The posterior perfusion pressure at 24 h and the plantar flexion after 4 weeks in both, peak torque (R = -0.73, p =< 0.05) and mean power (R = -0.7, p =< 0.05) displayed a significant correlation.

**Conclusion:**

The functional relationship between the decrease in intramuscular perfusion pressures and muscle performance in the early rehabilitation period indicate a causative and prognostic role of early posttraumatic microcirculatory derangements and skeletal muscle function. Therapeutic concepts aimed at effective muscle recovery, early rehabilitation, and decreased secondary tissue damage, should consider the maintenance of an adequate intramuscular perfusion pressure.

## Introduction

The severity of soft tissue trauma and the degree of secondary tissue damage, has a fundamental impact on the mid- and longterm prognosis of complex injuries to the extremities [1-3]. The extent of soft tissue injury is a result of both the direct tissue destruction by the trauma and the closely associated microvascular dysfunction and inflammatory response, as a secondary consequence to the initial trauma [4,5]. Derangements in capillary and nutritive perfusion, along with endothelial dysfunction, aggravates tissue oedema and intramuscular compartment pressures [6,7]. In turn, an increased compartment pressure beyond a critical threshold (acute compartment syndrome) deteriorates the nutritive perfusion by external capillary compression and restricts oxygen delivery. This causes tremendous pain and finally converges into a fatal vicious circle, of ischemia, inflammation and irreversible damage to vital neuromuscular structures [6,8,9]. Based on these underlying pathomechanisms, the established treatment for acute compartment syndrome includes an emergency fasciotomy, allowing the intramuscular pressure to decline. Therefore, in normotonic individuals, compartment pressure monitoring is recommended in order to anticipate the transition from impending, to the manifestation of compartment syndrome [8,10-12].

Among other factors, complete restitution of skeletal muscle contraction force, and the restoration of intramuscular energy resources are major determinants for the outcome. These influence the return of muscle function and determine the speed and success of rehabilitation. In particular, the direct impact of secondary fracture-associated soft tissue damage on long-term isokinetic skeletal muscle performance is only partly understood. Therefore, this study was aimed to quantitatively analyze the effect of soft tissue injury after isolated tibial fracture on the skeletal muscular outcome. This was assessed by measuring of the intramuscular perfusion pressure and the post-traumatic isokinetic muscle performance and recovery.

## Methods

### Study population and inclusion criteria

Between June 2004 and May 2005, 20 patients with isolated unilateral, solitary closed and open fractures of the tibia diaphysis, were prospectively studied (8 female, 12 males). The average age was 42 years (range: 25 to 65). All the in- and exclusion criteria were preset in a prospective study design prior to enrolment of patients. Due to the temporal profile of posttraumatic increase in tissue pressure, patients were only included if surgical treatment (closed or open reduction with internal or external fixation), started within the first 24 hours after trauma. Previous studies have demonstrated that the temporal profile of increase in intramuscular pressure in response to soft tissue trauma and/or fracture peaks within the first 24–48 hours [13,14]. In order to include maximum increase in intramuscular pressure and to correlate these changes to later muscle function, patients with trauma more than 24 hours ago, i.e. who possibly have already passed the maximum peak pressure, were excluded and not studied for tissue pressure monitoring. Before surgery, a time exposure was necessary in order to obtain both, a focused history from the patient, and to perform an appropriate examination to exclude additional injuries. Also, for the premedication procedure, in order to obtain written informed consent, and to organise surgical-capacity, additional time was necessary.

Patients with closed Tscherne G3- and open Gustilo typ IIIB/C soft tissue damage, i.e. with impending/manifest compartment syndrome or traumatic ischemia were not entered into the study, as the often subsequently performed emergency fasciotomy and compartment decompression does not allow a valid intramuscular pressure measurement. Patients with an age of less than 18 years, or patients with multiple life-threatening injuries (polytrauma), or traumatic brain injury (no written consent available), additional fractures of the ipsi- and/or contralateral extremity, or patients who developed manifest compartment syndrome requiring fasciotomy within the first 24 hours, were also excluded. Due to the increased risk of progressive hematoma and bleeding by percutaneous insertion of the microsensor probe, patients with blood coagulation disorders and/or anticoagulative medication were not enrolled into the study. Exclusion and inclusion criteria are summarized in Table 1.

**Table 1:** Peselected exclusion and inclusion criteria.

Exclusion criteria	Inclusion criteria
Age < 18 years	Written informed consent
Tscherne G3 or Gustilo Typ IIIB/C injuries	Tscherne G0/G1/G2 or Gustilo I°/II°/IIIa° injuries
Multiple life-threatening injuries (polytrauma)	Age > 18 years
Traumatic brain injury	No previous inury of the fracture site
Additional fractures of the ipsi- and/or contralateral extremity	Mono- injury
Manifested compartment syndrome	Surgical treatment within the first 24 h
Blood coagulation disorders	
Anticoagulative medication	

The criteria to plan surgical treatment followed the guidelines of the AO foundation [15]. Decision was made on the basis of clinical representation and the x-ray pictures. An informed written consent was obtained prior to participation in this study.

### Fracture classification

Fractures were classified according to the AO classification of long bones [15]. Soft tissue trauma was quantified by the Gustilo classification for open, and the Tscherne classification for closed fractures [16,17]. Patients with closed tibial fractures, with a Tscherne grade of C0, C1 and C2, and patients with a Gustilo grade of I to IIIA, were all included. Both the classification, and the treatment procedures of all patients, was evaluated by the senior author, who was blinded to the results of pressure measurement. All patients received standardized postoperative care, i.e. NSAR-medication, cryotherapy and immobilization for the first 24 hours.

### Pressure parameters

Intramuscular compartment pressure (IMP) recordings were assessed prior to surgery, directly postoperatively, 2, 4, 6, 8, 10, 12, 16, and 24 hours after surgery in the anterior (IMP_ant_) and deep posterior compartment (IMP_post_). Therefore, a CODMAN^® ^microsensor (0.7 mm outside diameter, Johnson & Johnson Professional, Inc., Raynham, MA, USA) was used, placed at the level of the fracture line.

Systolic, diastolic and mean arterial blood pressures (MAP) were monitored over 24 hours after trauma. The intramuscular perfusion pressure (PP) was calculated from the difference of mean arterial blood pressures, and the compartment pressure (PP_ant/post _= MAP - IMP_ant/post_). (As the blood pressure may change in response to local or multiple trauma, continuous monitoring of perfusion pressure, i.e., the difference between the mean arterial and venous pressure at the end of the capillary, has been proven to be a more valid adjunct in decision making for an early decompression [8,18].)

### Clinical appearance and blood parameters

Throughout the entire study period and postoperative course, clinical signs of a compartment syndrome were monitored continuously. The diagnosis of acute compartment syndrome of the thigh, was based on the diagnostic criteria previously described for acute compartment syndrome [19,20]. Diagnostic symptoms included thigh pain out of proportion to the injury, massive swelling and induration of the involved compartment, an increased thigh circumference, local pain that was aggravated by passive muscular stretch, weakness of the involved thigh muscles, or sensory or motor deficits in the anatomic distribution of the nerves contained in the involved compartment.

Serum levels of creatine kinase (CK), myoglobin, C-reactive protein (CRP), white blood cell count (WBC), haemoglobin (Hb) and haematocrit (Hct), were determined pre-operatively, one and four days after surgery.

### Muscular function

Muscle function was assessed using a Biodex dynamometer (Biodex Medical Systems Inc, New York, USA). Isokinetic peak torque and the mean power (considered as the endurance parameter) of the ankle joint in dorsiflexion and plantar flexion, were determined after 4 and after 12 weeks following injury. Peak torque was measured by five repetitions at a slow speed, (60°/s) while the mean power was assessed using 10 repetitions at an increased speed (120°/s). These tests were performed for both the uninjured, and the injured limb. Determined functional parameters for the uninjured limb were considered to be the patients individual muscle strength. Muscle function of the injured limb was expressed as a percentage of the uninjured one. All kinematical tests, were carried out by a research physiotherapist, who was blinded to the underlying compartment and perfusion pressure values

### Statistical analysis

The Kruskal-Wallis, Wilcoxon rank-sum, and Spearman's rank correlation coefficient, were used for statistical analysis. A significance was specified for a p value lower than 0.05 for all statistical test methods.

## Results

### Patient characteristics and distribution to the fracture classification

The patient characteristics and results of the AO fracture classification are shown in table 2. According to the underlying type and meta-/diaphyseal localization of the fracture, (based on the guidelines of the AO foundation), fourteen patients were treated with an intramedullary interlocking nail (Expert Tibial Nail, ETN, Synthes, Oberdorf, Switzerland). One was treated with an external fixator, and in five patients, ostheosynthesis was performed using percutaneously inserted angular stable plates, (LISS or Locking Compression Plates, LCP, Synthes, Oberdorf, Switzerland).

**Table 2:** Demographic Characteristics and Injury Patterns

Patient	Age, sex	Side	Aetiology	AO Classification	Tscherne classification (for closed fractures)	Gustilo classification (for open fractures)	Treatment method
1	47, m	Left	Traffic accident	42-B2	G1		nailing
2	25, f	Right	Traffic accident	42-C2		II°	nailing
3	35, m	Right	Traffic accident	43-A2	G2		ORIF
4	26, f	Right	Traffic accident	42-B2	G1		nailing
5	37, f	Left	Sport accident	42-C1		I°	nailing
6	48, f	Right	Traffic accident	42-B2		I°	nailing
7	54, f	Left	Fall	42-B3		II°	nailing
8	45, m	Left	Traffic accident	43-C1		II°	ORIF
9	63, m	Left	Fall	42-B2	G2		nailing
10	39, m	Right	Industrial accident	43-A1	G2		ORIF
11	42, m	Right	Traffic accident	42-B2	G2		nailing
12	28, m	Left	Sport accident	41-C1		I°	ORIF
13	40, m	Right	Traffic accident	42-C2		II°	nailing
14	23, m	Left	Sport accident	42-B2		I°	nailing
15	21, m	Left	Traffic accident	42-B2	G2		nailing
16	60, m	Right	Fall	43-C3		II°	ORIF
17	54, f	Left	Traffic accident	42-B3		II°	nailing
18	65, f	Right	Traffic accident	42-C2		IIIA°	external Fixateur
19	42, f	Right	Traffic accident	42-B1	G1		nailing
20	37, m	Right	Traffic accident	42-A2	G1		nailing

### Clinical appearance and blood parameters

None of the 20 investigated patients developed a clinically manifest compartment syndrome during the study period. Neither clinical suspicion, nor relevant persistent elevations of compartment pressures exceeding generally accepted limits [21], were found.

A positive correlation was shown, between the increase of serum levels of creatine kinase, and the perfusion pressure in the posterior compartment, 24 hours postoperatively (R = 0.61; p = 0.08). When studied, no further significant correlations were found, between perfusion pressure values, and serum levels of evaluated blood parameters.

### Intramuscular pressure parameters

**Figure 1:**
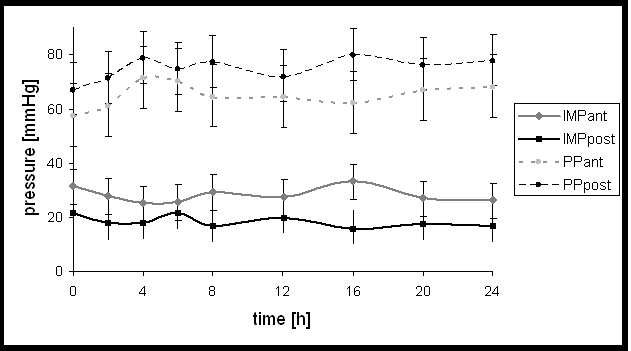
Course of compartment pressure (IMP) and perfusion pressure (PP) in the anterior- (ant) and deep posterior (post) compartment within the first 24 hours.

Figure 1 shows, the mean course of the intramuscular compartment pressure, and the muscular perfusion pressure in the anterior and deep posterior compartment, within the first 24 hours.

The IMP_ant _was significantly increased (P < 0.05) when compared to the IMP_post_, while the corresponding perfusion pressure was decreased (P < 0.05) (i.e. the perfusion pressure in the anterior compartment was significant decreased compared to perfusion pressure in the posterior compartment). In 6 patients, compartment pressures were temporary elevated over 40 mmHg, with an anterior pressure maximum of 63 mmHg after 2 hrs in one patient, which was measured in an anterior compartment. During the first 24 hours, all 20 Patients showed perfusion pressures higher than 40 mmHg.

### Muscular function

Mean deficit (%) in dynamometric Biodex measurements for peak torque, and mean power in dorsiflexion and plantar flexion after 4 and 12 weeks, respectively, are given in table 3.

**Table 3:** Dynamometric Biodex Measurements

	Peak torque		Mean power	
	
	Dorsiflexion	Plantar flexion	Dorsiflexion	Plantar flexion
Mean deficit (%) 4 weeks	50.1 ± 13.1^a^	61.3 ± 19.4	44.9 ± 17.7	55.3 ± 23.7
Mean deficit (%) 12 weeks	38.6 ± 20.1	45.7 ± 13	37.4 ± 16.3	27.9 ± 39.4

(Mean deficit (%) (to the uninjured side) in dynamometric Biodex measurements, for peak torque and mean power in dorsiflexion and plantar flexion after 4 weeks and 12 weeks. ^a^standard deviation)

**Figure 2:**
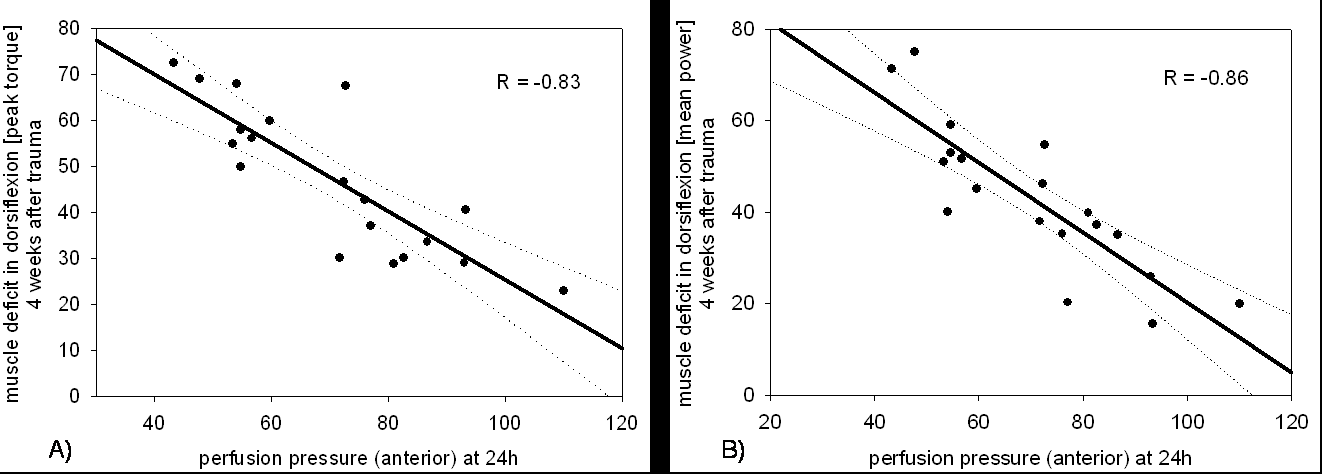
**Regression analysis of perfusion pressure on the muscle deficit after 24 hours, in dorsiflexion at 4 weeks after trauma.** (a) for the peak torque (R = -0.83; p < 0.05) and (b) for the mean power (R = -0.86; p < 0.05). Muscle deficit is given as a percentage of the non injured side, e.g. 80 percent means a 20 percent deficit.

**Figure 3:**
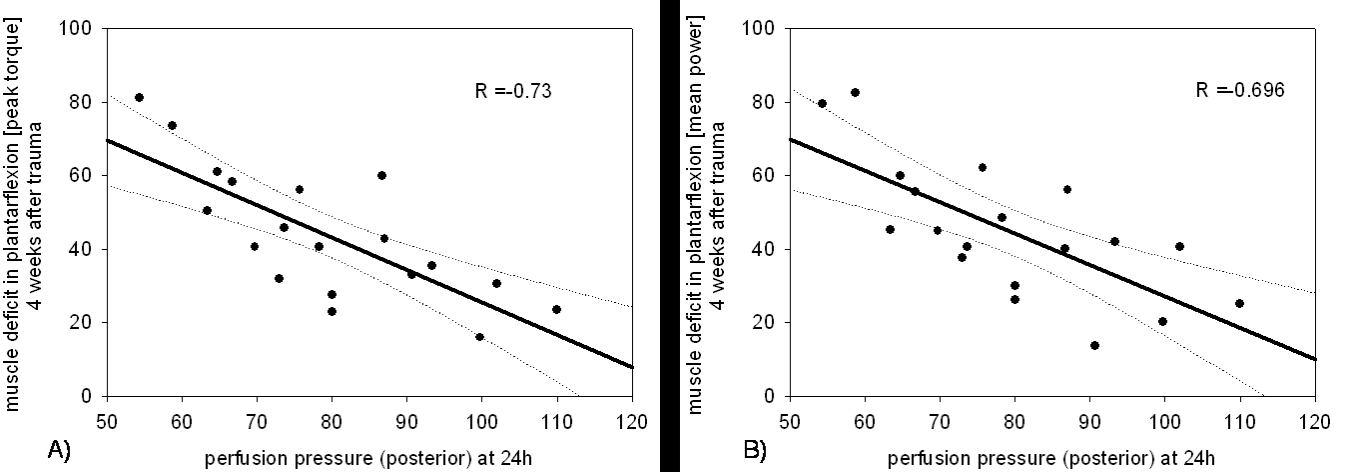
**Regression analysis of perfusion pressure on the muscle deficit after 24 hours, in plantar flexion at 4 weeks after trauma.** (a) for the peak torque and (b) for the mean power. (Rpeak = -0.73; Rmean = -0.696; p < 0.05) Muscle deficit is given as a percentage of the non injured side, e.g. 80 percent means a 20 percent deficit.

A significant correlation between the anterior perfusion pressure (PP_ant_), 24 hours postoperatively, and the dorsiflexion after four weeks was shown for both the peak torque (R = -0.83; p < 0.05) and the mean power (R = -0.86; p < 0.05) (Figure 2). A reduction of PP_post _after 24 hours, was also significantly correlated, to a uniformly decreased peak torque and mean power (R_peak _= -0.73; R_mean _= -0.696; p < 0.05) in plantar flexion after four weeks (Figure 3).

12 weeks following surgery no significant correlation was evident between perfusion pressure values and dorsi- or plantar flexion. The results are summarized in table 4 and 5.

**Table 4:** Biodex measurements (Dorsiflexion) after 4 and 12 weeks versus perfusion pressure in the anterior compartment at 24 hours

4 Weeks		12 Weeks	
Peak torque	Mean power	Peak torque	Mean power

R = -0.83	R = -0.86	R = -0.39	R = -0.48
p < 0.001	p < 0.001	p = 0.119	p = 0.07

(Biodex measurements (Dorsiflexion) after 4 and 12 weeks versus perfusion pressure in the anterior compartment at 24 hours. Statistical significance is given by p-values less than 0.05)

**Table 5:** Biodex measurements (Plantarflexion) after 4 and 12 weeks versus perfusion pressure in the posterior compartment at 24 hours

4 Weeks		12 Weeks	
Peak torque	Mean power	Peak torque	Mean power

R = -0.73	R = -0.696	R = -0.28	R = -0.39
p < 0.001	p < 0.001	p = 0.293	p = 0.121

(Biodex measurements (Plantarflexion) after 4 and 12 weeks versus perfusion pressure in the posterior compartment at 24 hours. Statistical significance is given by p-values less than 0.05)

## Discussion

In this present study, we were able to demonstrate a significant functional relationship between the trauma-induced reduction of perfusion pressure after 24 hours, in the anterior and posterior tibial compartment, and the skeletal muscle function in the early rehabilitation phase, i.e. 4 weeks postoperatively. The decrease in perfusion pressure after 24 hours, which was associated with a deficit in dorsiflexion and plantar flexion of the ankle joint after 4 weeks, indicates a causal-prognostic role of early microcirculatory deteriorations for a manifestation/development of skeletal muscle dysfunction, after four weeks post trauma.

Previous experimental and clinical studies have shown that tissue damage in response to soft tissue injury with endothelial dysfunction, edema, local inflammation and intramuscular pressure increase requires some time to develop [22]. Consequently, preceding studies of our group and others have shown that tissue pressure following trauma shows maximum peaks not before 24 hours after trauma [14,22]. Apart from these experimental reasons, we have also correlated the measured time points before 24 hrs. However, significant correlations were not found before 24 hours after surgery. This indicates that pressure increases at 24 hrs are most relevant and of prognostic importance for resultant muscle performance and muscle restoration 4 weeks after surgery. According to the limitation of the study period to 24 hrs, further conclusions about functional relationships between tissue pressure and muscle function could not be drawn.

In vivo analysis of microcirculation following soft-tissue injury demonstrated a interrelation between the severity of soft-tissue trauma and nutritive capillary derangements in skeletal muscle [14]. Progressive tissue damage, following severe soft-tissue injury, was shown to be a result of delayed and prolonged microvascular perfusion failure. These results imply that post-traumatic muscle dysfunction may in fact be caused by the direct trauma, although the extent of impairment seems mainly influenced by the degree of posttraumatic perfusion disturbance. Crisco et al. have investigated biomechanical, physiological and histological alterations in a gastrocnemius muscle contusion injury model, of male Wistar rats [23]. They also demonstrated a significant deficit in contractile function, in relation to the extent of contusion injury.

In addition, supporting the notion that the extent of muscle trauma is a limitating co-factor to posttraumatic muscle performance, Shaw and co-workers showed a significant relationship between the severity of tibial fractures, and the resulting rehabilitation time in football players [24]. It could also be observed, that fracture morphology, the presence of an open wound and the Tscherne grade of closed fractures correlated with regained muscle power [25]. Also, in addition to the severity of the initial injury, the patient's age seems to be one of the main factors influencing muscle recovery following diaphyseal tibia fractures [25,26]. The fact that in our study, no correlation between muscle recovery and age was found may be due both to the small variation in age of the included patients, with the oldest patient being 65 years, and the comparably small number of included patients.

Similar to our findings, Gaston et al. could show that muscle function of the ankle and subtalar joints, recover quickly from an initially low level [25]. They have further found, that the differences in muscle power caused by age, muscle damage, and the type of fracture, became more obvious not before 15 to 20 weeks. The fact that our study period was limited to 12 weeks, may explain why we did not detect differences, in the outcome which depended on age, or the type of fracture.

Our findings suggest that, the initial posttraumatic changes in microcirculation within the first 24 hours have a prognostic and predictive importance for muscle recovery at 4 weeks after surgery. Early muscle recovery is in turn, an absolute prerequisite for rapid mobilization, and accelerated rehabilitation. In this context, effective treatment strategies after lower leg injuries have to ensure the restitution of nutritive perfusion, and the maintenance of sufficient perfusion pressure, in order to prevent subsequently impaired muscle performance and delayed rehabilitation. The short immobilization period for the first couple of days is beneficial in providing a sufficient phagocytosis of necrotized tissue and granulation tissue formation. However, for regeneration of myofibers and capillary ingrowth, a specifically early mobilization procedure was shown to be essential [5,23,27,28]. Early, postoperative mobilization was introduced in 1954 [29]. Apart from these positive mobilization-associated effects of the regeneration of skeletal muscle morphology, biomechanical in vitro investigations, also demonstrated a faster return of muscle strength to the level of the uninjured contralateral muscle, following an active early mobilization [27].

Our results confirm that perfusion pressure (calculated from the difference of the mean arterial pressure and the compartment pressure) correlates significantly with the post traumatic muscle performance while absolute intracompartimental pressures alone did not. Perfusion pressure is, by taking into account the arterial blood pressure, i.e. the macrohemodynamic situation, a more valid parameter to reflect posttraumatic muscle tissue damage. As a result, an increased compartment pressure in combination with an adequate blood pressure appears to not be unavoidably related with a greater extent of muscle cell damage, risk of compartment syndrome, or an impaired post traumatic muscle performance. In our study, 6 patients had a temporary compartment pressure higher than 40 mmHg. In all of these patients, a sufficient perfusion pressure was calculated and existed. The later performed Biodex measurements in these patients corresponded to the perfusion pressure, while a relationship to compartment pressures was not shown. Despite an elevation in the compartment pressure, the evaluated peak torque and mean power results were in the range of the other patients. This notion is confirmed by an evaluation of skeletal muscle metabolism with nuclear magnetic resonance spectroscopy [30]. The authors demonstrated, that metabolic derangements mainly depend on the difference between MAP and compartment pressure, rather than on absolute compartment pressure [30]. It was shown, that a perfusion pressure of less than 40 mm Hg in bluntly traumatized muscle, was associated with tissue acidosis and ischemia. Again, investigating the relationship between compartment and perfusion pressure, Hartsock et al. demonstrated, that capillary perfusion in skeletal muscle is equally and profoundly impaired, either at a PP of 25.5 ± 14.3 mm Hg or a compartment pressure exceeding 60 mmHg [31]. In addition, Whitesides et al. were the first to recommend that differential perfusion pressure, as opposed to absolute intramuscular pressures, were of high importance [32]. This underlines the essential significance of local and distal tissue perfusion.

In a recent study, White et al. demonstrated, that a decrease of perfusion pressure to a lower limit of 30 mm Hg, and an elevated intramuscular pressure to an upper level of 70 mm Hg, is tolerated without significant adverse consequences [33]. Obviously, a parallel/simultaneous elevation of both the diastolic blood and the intramuscular perfusion pressure, maintains an adequate capillary perfusion. Thus enables the tissue to tolerate elevated compartment pressures. Consideration should be given to polytraumatized patients, where possibly prolonged periods of insufficient circulation coupled with a depressed blood pressure and an inadequate oxygenation, may lead to a shift of the critical threshold of tissue tolerance, into decreased compartment pressures. However, the combination of clinical awareness, and the continuous differential perfusion pressure monitoring, as based on our experience and that of other authors [34,35], is a much more effective, specific and reliable method in detecting a subsequent compartment syndrome, as opposed to just measuring absolute intracompartimental pressure values. Furthermore, the measurement of intramuscular pressure alone, as a criterion for fasciotomy, has a lower specificity, and was shown to result in an unnecessarily high fasciotomy rate and an increased rate of associated short- and long term complications [36].

## Conclusion

We were able to show a significant correlation between the perfusion pressure after 24 hours and the functional outcome in muscle performance after 4 weeks. There was no correlation between muscle function and the intracompartimental pressure itself. Alterations in muscle perfusion, caused by primary and secondary soft-tissue damage were responsible for the substantial muscle dysfunction seen for up to 4 weeks following trauma. Obviously, monitoring perfusion pressure is far more superior and sensitive, in the assessment of post-traumatic effects on muscle performance and recovery. Therefore, effective treatment strategies must be made to ensure the restitution of nutritive perfusion and sufficient perfusion pressure. This is in order to prevent future deficits in muscle performance, and a delayed rehabilitation.
